# Supplementary material for: Integrating genomic structural equation modeling and experimental validation to unravel the genetic basis of male genital lichen sclerosus
Source: Front Immunol. 2026 Jul 16;17:1857945. doi: 10.3389/fimmu.2026.1857945 (PMC13435250; doi:10.3389/fimmu.2026.1857945)
Supplement: Supplementary file 6 [file SupplementaryFile5.zip › result/fuma_PSYCH_factor_sCCA1_focus.chr9.chr9.72753755.74253040.pop1.pdf]

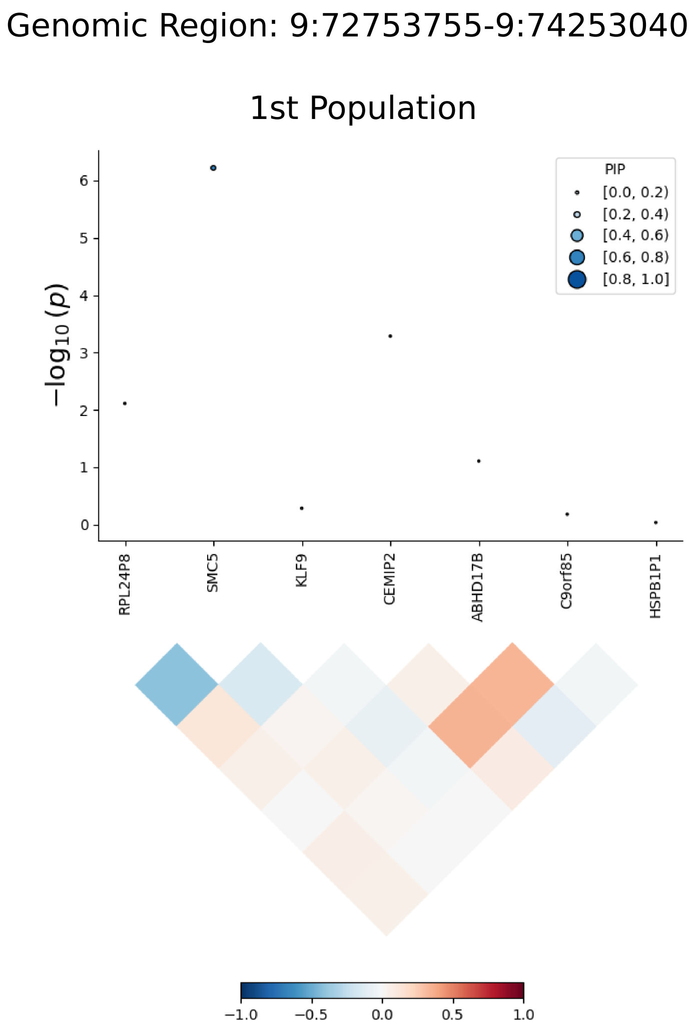

Genomic Region: 9:72753755-9:74253040
1st Population
$-\log_{10}(p)$
PIP
[0.0, 0.2)
[0.2, 0.4)
[0.4, 0.6)
[0.6, 0.8)
[0.8, 1.0]
RPL24P8
SMC5
KLF9
CEMIP2
ABHD17B
C9orf85
HSPB1P1
−1.0
−0.5
0.0
0.5
1.0
